# 超高效液相色谱-四极杆/线性离子阱质谱法同时确证和测定牛源性食品中4种新型异噁唑啉类兽药残留

**DOI:** 10.3724/SP.J.1123.2024.11012

**Published:** 2025-09-08

**Authors:** Cheng YANG, Weixia ZHU, Yafeng LIU, Wei WEI, Fang ZHAO, Kai HU, Wenjie ZHAO

**Affiliations:** 1.郑州海关技术中心，河南 郑州 450003; 1. Zhengzhou Customs District，Zhengzhou 450003，China; 2.河南中医药大学，中医药科学院，河南 郑州 450046; 2. Academy of Chinese Medical Science，Henan University of Chinese Medicine，Zhengzhou 450046，China; 3.河南工业大学化学化工学院，河南 郑州 450001; 3. School of Chemistry and Chemical Engineering，Henan University of Technology，Zhengzhou 450001，China

**Keywords:** 固相萃取, 超高效液相色谱-四极杆/线性离子阱质谱, 异噁唑啉类药物, 氟雷拉纳, 牛源性食品, solid phase extraction （SPE）, ultra-high performance liquid chromatography-quadrupole/linear ion trap mass spectrometry （UHPLC-Q/Trap MS）, isoxazoline drugs （ISOs）, fluralaner, bovine-origin foods

## Abstract

异噁唑啉类药物（ISOs）是一类含有N和O原子的五元杂环类化合物，它们能够抑制*γ*-氨基丁酸门控氯离子通道，被广泛用于家禽寄生虫病的治疗。人类摄入动物源性食品是接触ISOs的重要途径，为解决动物源性食品中ISOs残留带来的安全问题，本研究建立了一种针对牛源性食品（包括牛奶、牛肉和牛肝）中4种新型ISOs（氟雷拉纳、洛替拉纳、沙罗拉纳和阿福拉纳）的超高效液相色谱-四极杆/线性离子阱质谱（UHPLC-Q/Trap MS）分析方法。样品先经乙腈提取，再用PRiME HLB固相萃取小柱净化；以5 mmol/L乙酸铵水溶液和乙腈作为流动相，经Shim-pack GIST C18-AQ （100 mm×2.1 mm， 2.7 μm）色谱柱分离后，在多反应监测（MRM）模式下，采用信息依赖性采集（IDA）、增强子离子扫描（EPI）和谱库检索进行分析，并使用外标法进行定量。结果显示，4种ISOs在各自的质量浓度范围内线性关系良好，相关系数（*r*）均≥0.993 6，检出限（LOD）和定量限（LOQ）分别为0.2~0.5 μg/kg和0.5~1.0 μg/kg；在低、中、高3个加标水平（1、2、10 μg/kg）下，4种ISOs的回收率为67.6%~118.9%，相对标准偏差（RSD）为2.0%~20.0%。此外，本研究通过MRM-IDA-EPI结合谱库检索对目标化合物进行定性筛查分析，依据色谱保留时间和EPI碎片离子等信息对目标化合物进行双重定性，提高了定性分析的准确性，有效排除了假阳性结果的干扰。该方法具有LOD低、回收率良好等特点，且操作简单、快速，具备高灵敏度和高准确度，能够实现牛源性食品中新型ISOs残留的定性和定量分析。

## Abstract (en)

Isoxazoline drugs （ISOs） are a class of five-membered heterocyclic compounds containing N and O atoms. They can inhibit *γ*-aminobutyric acid gated chloride channels and are widely used in the treatment of parasitic diseases in poultry. The intake of animal-derived foods by humans is an important way to come into contact with ISOs. Excessive use of ISOs can lead to their residues in animal-derived foods， thereby threatening human health and causing neurotoxicity and hepatotoxicity. To address the safety issues caused by ISO residues in animal-derived foods， an ultra-high performance liquid chromatography-quadrupole/linear ion trap mass spectrometry （UHPLC-Q/Trap MS） analytical method for four novel ISOs （fluralaner， sarolaner， afoxolaner， lotilaner） in bovine-origin foods （including milk， beef and bovine liver） was established. The sample was first extracted with acetonitrile and then purified with PRiME HLB solid phase extraction （SPE） column. Using 5 mmol/L ammonium acetate aqueous solution and acetonitrile as the mobile phase， after separation by Shim-pack GIST C18-AQ （100 mm×2.1 mm， 2.7 μm） chromatography column， the analysis was carried out in the multi-reaction monitoring （MRM） mode by information-dependent acquisition （IDA）， enhanced product ion scanning （EPI） and spectral library retrieval， and quantification was performed using the external standard method. The results showed that the four ISOs had good linear relationships within their respective mass concentration ranges. The correlation coefficients （*r*） were all ≥0.993 6， and the limits of detection （LODs） and quantification （LOQs） were 0.2-0.5 μg/kg and 0.5-1.0 μg/kg， respectively. Under the low， medium and high spiked levels （1， 2 and 10 μg/kg）， the recoveries of the four ISOs ranged from 67.6% to 118.9%， and the relative standard deviations （RSDs） ranged from 2.0% to 20.0%. In addition， in this study， qualitative screening and analysis of the target compounds were conducted through MRM-IDA-EPI combined with spectral library retrieval. Dual qualitative analysis of the target compounds was carried out based on information such as retention time and EPI fragment ions， which improved the accuracy of qualitative analysis and effectively eliminated the interference of false positive results. This method features low LODs and good recoveries. It is also simple and rapid to operate， with high sensitivity and accuracy. It can achieve qualitative and quantitative analysis of new ISOs residues in bovine-origin foods. This study can provide technical support for food safety agencies to implement preventive measures against new ISOs in animal foods.

寄生虫病是由节肢动物、蠕虫和原虫等寄生虫引发的一种常见动物疾病，其致病机理较为复杂。长期以来，除虫菊酯类、有机磷类和新烟碱类等药物一直是寄生虫防治的主要药物^［1-3］^。然而，由于这些药物在畜牧业中广泛使用，出现了高残留、高毒性以及存在副作用等问题，因而受到限制或被禁用。异噁唑啉类药物（isoxazoline drugs，ISOs）是一类含有N和O原子的五元杂环化合物，主要通过干扰存在于脊椎动物和无脊椎动物体内的*γ*-氨基丁酸门控氯离子通道来发挥作用。这类药物具有良好的生物活性和出色的安全性等优点，近年来作为驱虫剂被应用于畜牧、家禽和宠物等动物寄生虫的防治工作中，并且与现有的杀虫剂没有明显的交叉抗药性^［4-6］^。然而，过量使用ISOs会导致其在动物源性食品中残留过量，对人类健康构成威胁，引发神经毒性和肝毒性^［7］^。目前，欧洲药品管理局、澳大利亚农药和兽药管理局等国际监管机构已制定规定，明确氟雷拉纳在家禽中的最大残留限量为0.06~1.3 mg/kg^［8］^。现阶段，对于新型ISOs的研究主要集中在工业合成和作用机理等方面，而有关动物源性食品中ISOs残留检测的研究相对较少。因此，迫切需要开发一种动物源性食品中ISOs残留的检测方法。

超高效液相色谱-串联质谱（UHPLC-MS/MS）、液相色谱（HPLC）和气相色谱-质谱（GC-MS）等技术已成功应用于兽药残留的分析工作中^［9-11］^。其中，UHPLC-MS/MS技术凭借其高效的分离能力、高通量、高灵敏度以及应用范围广等特性，成为兽药残留研究中极具优势的分析技术。例如，夏玉吉等^［12］^基于UHPLC-MS/MS技术，建立了猪肝中沙罗拉纳残留的分析方法。中华人民共和国农业农村部公告第657号规定，采用UHPLC-MS/MS测定动物源性食品（鸡肉、鸡蛋、鸡肝和鸡肾）中的氟雷拉纳残留^［13］^。超高效液相色谱-四极杆/线性离子阱质谱（UHPLC-Q/Trap MS）的多反应监测-信息依赖性采集-增强子离子扫描（MRM-IDA-EPI）模式具备强大的定性与定量功能。通过一针进样，可同时获取MRM定量数据以及高灵敏度的二级碎片 EPI 全谱图。结合谱库检索，能够准确地对化合物进行定性，该检测模式为动物源性食品中兽药残留的定性分析提供了良好的技术支撑^［14］^。郑姝宁等^［15］^利用UHPLC-Q/Trap MS技术建立了蔬菜中176种农药残留的快速筛查分析方法，在MRM-IDA-EPI检测模式下，176种农药与数据库的匹配值均大于67%。Zhou等^［16］^利用UHPLC-MS/MS技术开发了一种灵敏度高且选择性好的方法，用于生物样品中9种亚硝胺的检测，同时采用MRM-IDA-EPI扫描模式来消除假阳性结果，整个检测过程具有耗时短、样品制备简便等特点。Zhou等^［17］^采用UHPLC-Q/Trap MS法，对15种氨基甲酸酯农药残留进行了系统分析，并利用EPI图谱对可疑样品中的氨基甲酸酯农药进行筛查。然而，目前尚未见有研究采用该技术对动物源性食品中ISOs的残留进行定性筛查和定量分析。

目前，巴西和澳大利亚等国外相关权威机构已规定氟雷拉纳等新型ISOs在牛源性食品中的残留限量标准，而我国尚无相关限量规定，且尚未检索到牛源性食品中ISOs检测的相关报道。

本研究将固相萃取技术与UHPLC-Q/Trap MS技术结合，利用MRM-IDA-EPI扫描模式，建立了一种可同时测定牛源性食品（牛奶、牛肉和牛肝）中4种新型ISOs（氟雷拉纳、洛替拉纳、沙罗拉纳和阿福拉纳）的分析方法。该方法能够同时对新型ISOs进行定性和定量分析，可作为日常监测的有效手段，为动物源性食品中ISOs的残留检测提供技术支撑。

## 1 实验部分

### 1.1 仪器、试剂与材料

AB 4500 QTRAP液相色谱-质谱联用系统（美国AB SCIEX公司），KQ-300DE型数控超声波清洗器（昆山市超声仪器有限公司），WIGGENS Vortex 3000-Elite涡旋振荡器（维根技术有限公司），24位固相萃取装置（上海安谱实验科技股份有限公司），CF15RXII离心机（日本Hitachi公司），DS-1高速组织捣碎机（上海欧盟实业有限公司），112N-EVAP112氮吹仪（美国Organo-mation associates公司），PRiME HLB固相萃取柱（60 mg/3 mL，美国Waters公司）。0.22 μm有机滤膜（天津津腾公司）。

氟雷拉纳、阿福拉纳、洛替拉纳和沙罗拉纳（对照品，纯度均≥99%，加拿大TRC公司）；乙酸铵、乙腈（ACN）、甲醇（MeOH）和甲酸（FA）（色谱级，美国Fisher公司），25%~28%氨水（分析纯，上海麦克林生化科技股份有限公司）。实验用超纯水（18.2 MΩ⋅cm）由Milli-Q纯化系统（美国Millipore公司）制备。

牛奶、牛肉和牛肝样品购自郑州市不同地区的超市或市场。将牛肉和牛肝样品储存于-20 ℃冰箱中备用，牛奶样品不做处理储存于4 ℃冰箱中备用。

### 1.2 溶液的配制

精密称取4种ISOs对照品各1.0 mg，用乙腈稀释至10 mL，配制成质量浓度为100 mg/L的单标储备液，并储存于-20 ℃冰箱中。分别准确吸取适量的单标储备液，用乙腈混合稀释，配制成4种ISOs质量浓度均为1 mg/L的混合标准中间液。用5 mmol/L乙酸铵水溶液-乙腈（1∶1，v/v）逐级稀释混合标准中间液，配制成质量浓度分别为1、2、5、10、50、100、200、500 μg/L的混合标准工作液，现用现配。

### 1.3 样品前处理

将牛肉和牛肝样品解冻至室温后，各称取200 g，进行匀浆处理，并将均质后的牛肉和牛肝样品储存于-18 ℃冰箱中备用。将匀浆后的牛肉和牛肝样品解冻至室温，随后各称取2.00 g（精确至0.05 g）牛肉和牛肝样品及牛奶样品，分别置于50 mL离心管中，加入5 mL乙腈，涡旋振荡1 min，超声提取10 min，之后在5 000 r/min下离心5 min，取出上清液；将上清液用超纯水定容至10 mL，待净化。将全部待净化样液过PRiME HLB固相萃取小柱，之后分别用1 mL超纯水和1 mL 10%甲醇水溶液淋洗固相萃取小柱，弃去淋洗液后，用2 mL 5%氨水甲醇进行洗脱，收集洗脱液，置于40 ℃下氮吹至近干；最后，用5 mmol/L乙酸铵水溶液-乙腈（1∶1，v/v）定容至1 mL，再过0.22 µm滤膜，待UHPLC-Q/Trap MS分析。若样品中待测物含量不在标准曲线的线性范围，则进行适当稀释后再检测。

### 1.4 仪器条件

#### 1.4.1 色谱条件

Shim-pack GIST C18-AQ色谱柱（100 mm×2.1 mm， 2.7 μm）；柱温：50 ℃；进样量：10 μL。流动相由5 mmol/L乙酸铵水溶液（A）和乙腈（B）组成，流速为300 µL/min。梯度洗脱程序：0~4.0 min，50%B；4.0~4.1 min，50%B~75%B；4.1~7.0 min，75%B；7.0~7.1 min，75%B~50%B；7.1~12.0 min，50%B。

#### 1.4.2 质谱条件

离子源：电喷雾电离（ESI）源，负离子模式；毛细管电压：5 500 V；入口电压：10 V；雾化气压力：345 kPa；气帘气压力：172 kPa；加热辅助气压力379 kPa；离子源温度：550 ℃。4种ISOs的监测离子对及质谱参数等信息见表1。当MRM信号强度大于IDA所设定的阈值（1 000 cps）时，同时启动线性离子阱的EPI功能。EPI扫描参数设置如下：扫描范围为*m/z* 50~500，执行动态背景扣除，不排除前级离子，扫描速率为20 000 Da/s，碰撞电压（CE）为（35±15） eV。其他参数与MRM模式相同。

**表1:** 4种ISOs的保留时间和质谱参数

Compound	Retention time/min	Parent ion （*m/z*）	Product ions（*m/z*）	DP/V	CEs/eV
Sarolaner	6.99	579.0	147.7^*^， 508.7	120	30， 25
Fluralaner	7.14	554.0	534.0^*^， 514.0	120	25， 30
Lotilaner	7.71	594.0	180.9^*^， 160.8	120	28， 40
Afoxolaner	7.87	624.0	604.0^*^， 564.0	150	30， 35

∗ Quantitative ion. DP： declustering potential； CEs： collision energies.

### 1.5 EPI-MS数据库的建立

利用4种ISOs的混合标准工作液（100 μg/L）在MRM-IDA-EPI模式下对目标物进行检测，获取子离子扫描质谱图，并据此建立谱库，用于ISOs的定性检索。在SCIEX OS软件（3.4.5版，美国AB SCIEX公司）现有谱库的基础上，将目标物的分子式、CAS号、化学名称、结构式以及EPI扫描子离子质谱图等信息录入数据库，建立4种新型ISOs的EPI谱库。在实际样品检测过程中，若目标物与谱库比对所得分值≥85分，则判定样品中含有ISOs。

## 2 结果与讨论

### 2.1 质谱条件优化

4种ISOs的结构中均含有不同数量的有机氯原子，在进行质谱分析时，可能会出现多个同位素峰，这些同位素峰会对目标化合物的主信号峰造成干扰，进而影响信号的准确积分与识别以及对目标物的定性判断。因此，分析ISOs中有机氯同位素的分布情况，对于目标物的准确定量和定性具有重要意义。在采用ESI源对目标物进行全扫描（Scan）时，氟雷拉纳主要呈现出3个有机氯同位素峰，分别为M峰（分子离子峰，*m/z* 554.0）、M+2峰（*m/z* 556.0）和M+4峰（*m/z* 558.0），其质谱峰丰度比约为9∶6∶1，故选择与^35^Cl对应的M峰［M-H］^-^（*m/z* 554.0）作为氟雷拉纳的母离子。在沙罗拉纳的全扫描质谱图中，可观察到［M-H］^-^峰（*m/z* 579.0）及其同位素^37^Cl离子峰［M-H］^-^（*m/z* 581.0），且^35^Cl离子峰的信号强度大于^37^Cl离子峰的信号强度，故选择［M-H］^-^（*m/z* 579.0）作为沙罗拉纳的母离子。在阿福拉纳的质谱图中，观察到［M-H］^-^峰（*m/z* 624.0）及其同位素^37^Cl离子峰［M-H］^-^（*m/z* 626.0），其质谱峰丰度比约为3∶1，故选择［M-H］^-^（*m/z* 624.0）作为阿福拉纳的母离子。洛替拉纳含有3个有机氯原子，在质谱扫描时，主要出现*m/z* 594.0、*m/z* 596.0、*m/z* 598.0和*m/z* 600.0信号峰，分别对应M峰、M+2峰、M+4峰和M+6峰，它们的丰度比约为27∶27∶9∶1；其中，［M-H］^-^（*m/z* 594.0）与其同位素^37^Cl离子峰［M-H］^-^（*m/z* 596.0）均具有较好的质谱响应信号，但［M-H］^-^（*m/z* 594.0）的信号更稳定，故选择［M-H］^-^（*m/z* 594.0）作为洛替拉纳的母离子。

依据所选目标物的母离子，对子离子碎片进行优化，根据响应强度确定对应的子离子。在ESI^-^模式下，4种目标物均呈现出较好的响应强度，能够满足目标物的定量与定性分析需求。在质谱裂解过程中，4种ISOs均有一个或多个氟化氢（HF）脱落。从中选择丰度较高且干扰较小的两个产物离子，分别作为定性离子和定量离子。同时，对定性和定量离子对的去簇电压（DP）和碰撞能量（CE）进行优化，优化后得到的各目标物质谱参数见表1，4种ISOs可能的质谱裂解途径见附图1（www.chrom-China.com）。

### 2.2 EPI谱图分析和谱库匹配度验证

由于目标物在肉源性食品中的含量较低，在采用MRM模式进行分析时，容易受到基质干扰的影响，导致定性离子和定量离子的丰度比率易超出容许范围，进而影响定性判定，出现假阳性或假阴性的情况。MRM-IDA-EPI技术具有特有的信息依赖性复合全扫描功能，在该功能下，累积的离子在一定碰撞能量作用下会产生更多二级碎片离子，随后这些离子进入检测器扫描，从而获得EPI谱图。在MRM分析获取目标物的保留时间、峰高、峰面积和离子比率等信息的同时，通过EPI扫描获得二级质谱图，再将其与谱库进行检索对比，可对目标物进行双重鉴定。这种双重鉴定方式能够提高定性分析的准确性，更有利于对痕量目标物进行准确定性分析。在牛肉样品中添加含量为1.0 μg/kg的目标物，按1.3节方法进行前处理，然后对其进行MRM-IDA-EPI扫描检测，并将EPI谱图与谱库进行比对。如附图2所示，氟雷拉纳、阿福拉纳、洛替拉纳和沙罗拉纳的匹配值分别为99.3、99.6、99.5和98.9。这一结果表明，尽管实际基质样品中ISOs的含量较低，但仍可获得高灵敏度的EPI质谱图，从而确保定性结果的准确性。

### 2.3 前处理条件的优化

#### 2.3.1 提取溶剂的选择

ISOs含有C-F等非极性基团，极性较小，其油水分配系数（log *P*）为4.86~6.38。基于动物源性食品的特性和ISOs的溶解性，本研究考察了乙腈、乙腈-水（9∶1，v/v）、1%甲酸乙腈、甲醇、甲醇-水（9∶1，v/v）以及1%甲酸甲醇6种溶剂对4种ISOs的提取效果。如图1a所示，采用甲醇或甲醇-水（9∶1，v/v）进行提取时，提取效果均较差，4种ISOs的回收率分别为61.2%~97.3%和39.1%~86.9%。虽然添加1%甲酸能够明显提高甲醇对沙罗拉纳和氟雷拉纳的回收率，但对于阿福拉纳和洛替拉纳的提取效果仍不理想。当使用乙腈进行提取时，其提取效果明显优于甲醇，回收率为80.0%~105.5%，能够满足GB 2763-2021^［18］^中关于兽药残留分析的要求。同时，与单独使用乙腈相比，1%甲酸乙腈和乙腈-水（9∶1，v/v）对4种ISOs的提取效果并无明显提升。因此，本研究选择乙腈作为提取溶剂。

**图1:**
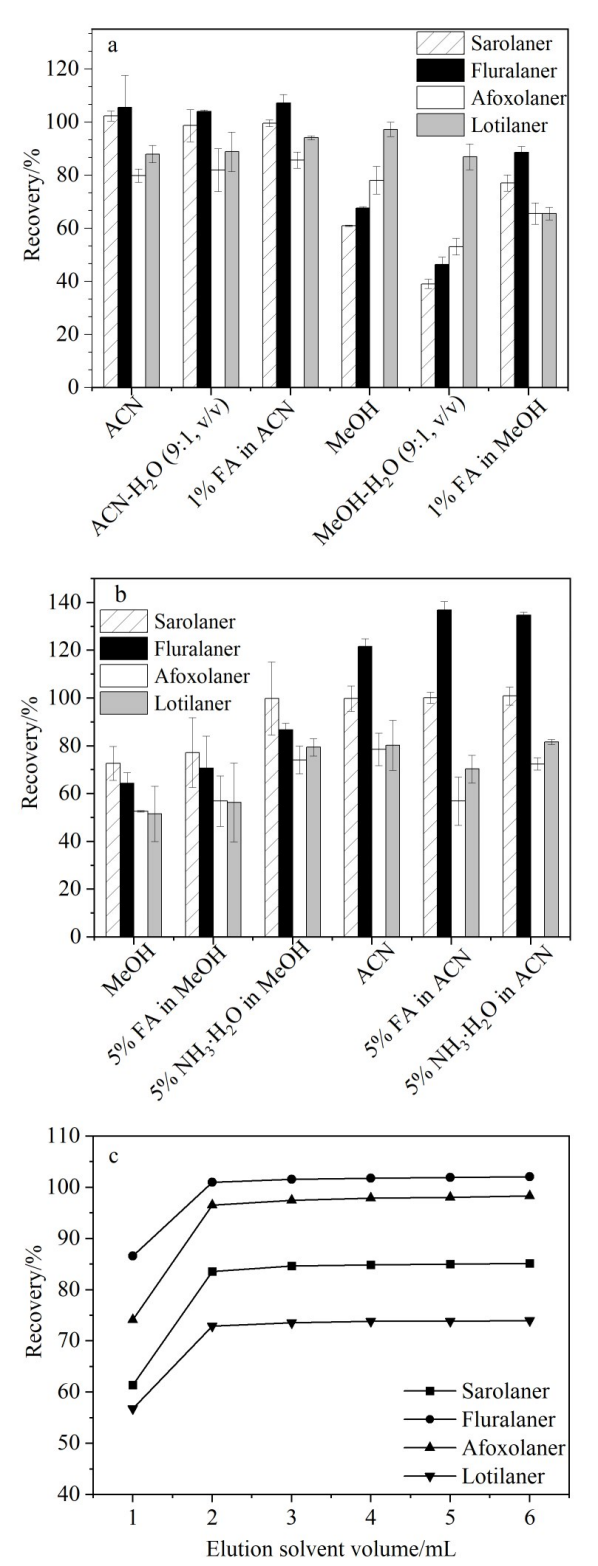
（a）提取溶剂种类、（b）洗脱溶剂种类（*n*=3）及（c）洗脱溶剂体积对4种ISOs回收率的影响

#### 2.3.2 固相萃取柱的选择

本研究比较了Oasis HLB （60 mg/3 mL）、Oasis PRiME HLB （60 mg/3 mL）、Oasis WAX（60 mg/3 mL）、Oasis WCX（60 mg/3 mL）、Oasis MCX（60 mg/3 mL）、Bond Elut C18（150 mg/6 mL）和Sep-Pak NH₂ （500 mg/6 mL）7种吸附性能不同的固相萃取小柱对4种ISOs的净化效果。结果表明，当使用WAX小柱和Sep-Pak NH₂小柱进行净化时，回收率明显偏高（80.1%~151.3%），表明二者对ISOs的净化效果不佳。WCX小柱和MCX小柱的净化效果差异较小，不过二者对沙罗拉纳的净化效果均不理想，平均回收率分别为60.4%和57.4%。PRiME HLB小柱、Oasis HLB小柱和C18小柱的净化效果较为理想，能够满足日常分析的需求，但在填料用量相同的情况下，PRiME HLB萃取小柱和Oasis HLB萃取小柱具有更大的吸附容量。与Oasis HLB小柱相比，PRiME HLB小柱对沙罗拉纳的净化效果更优。此外，PRiME HLB小柱将目标物分离与样品净化整合为一个步骤，还能较好地去除样品基质中的蛋白质、盐类、脂肪和磷脂等干扰物质，有利于对高脂肪和高蛋白的动物源性食品进行分析。因此，本实验最终选用PRiME HLB固相萃取小柱对样品进行净化。

#### 2.3.3 洗脱溶剂种类及用量的优化

本实验考察了甲醇、5%甲酸甲醇、5%氨水甲醇、乙腈、5%甲酸乙腈和5%氨水乙腈对4种ISOs的洗脱效果。结果如图1b所示，当使用乙腈作为洗脱溶剂时，氟雷拉纳的回收率过高（121.7%），不符合兽药分析标准。使用5%甲酸乙腈和5%氨水乙腈作为洗脱溶剂时，氟雷拉纳的回收率结果仍偏高，未得到改善。使用甲醇为洗脱溶剂时，4种ISOs的回收率为51.5%~72.7%，而当添加5%甲酸或5%氨水后，洗脱效果明显改善；相比之下，5%氨水甲醇的洗脱效果更为理想，4种ISOs的回收率更高（71.1%~99.8%）。因此，本实验选择使用5%氨水甲醇对目标物进行洗脱。此外，实验对洗脱溶剂的用量（1、2、3、4、5、6 mL）进行了优化，结果如图1c所示。当洗脱溶剂体积从1 mL增加至2 mL时，回收率增加；当洗脱溶剂体积超过2 mL后，4种ISOs物的回收率趋于稳定，因此最终确定5%氨水甲醇的体积为2 mL。在最优实验条件下，4种ISOs的MRM色谱图如图2所示。

**图2:**
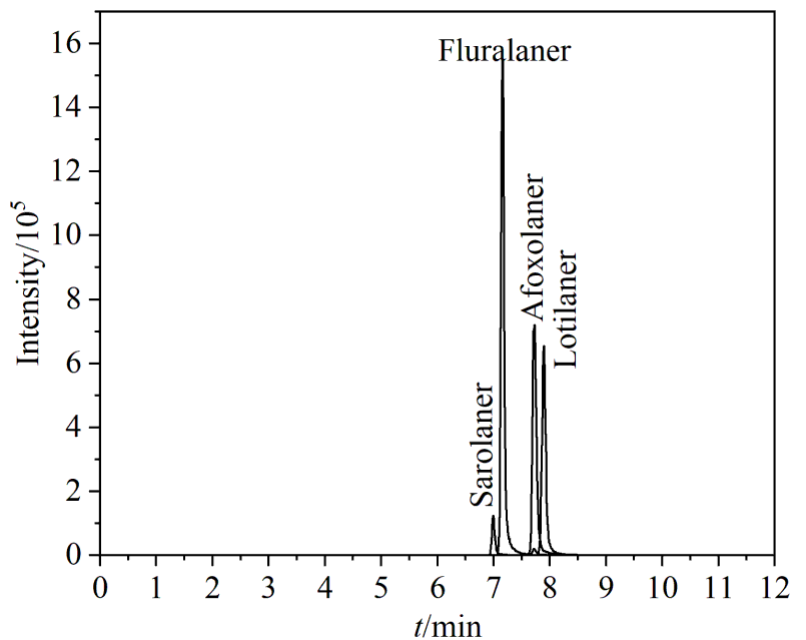
最优实验条件下4种ISOs的MRM色谱图

### 2.4 基质效应（ME）

样品中除目标分析物以外的成分称为基质，在使用UHPLC-Q/Trap-MS分析样品时，基质中目标分析物的信号响应可能会被抑制或增强，进而影响方法的重现性和准确性^［19，20］^。本文以3种空白牛源性食品（牛奶、牛肉和牛肝）作为样品基质，按1.3节方法进行前处理，获得空白基质提取液；利用3种空白基质提取液分别配制系列质量浓度的基质匹配混合标准溶液，同时用乙腈配制相同质量浓度的溶剂混合标准溶液。依据ME=（基质匹配标准曲线斜率/溶剂标准曲线斜率-1）×100%来评估不同样品基质中的基质效应。当ME=0时，表示无基质效应；当ME>0时，表示存在基质增强效应；当ME<0时，表示存在基质抑制效应。进一步地，当|ME|≤20%时，说明基质效应较弱，在实际检测中可忽略不计；当20%<|ME|<50%或|ME|≥50%时，说明存在中等程度基质效应或较强基质效应，此时应采取相应措施来减少基质效应对目标物检测的干扰。如图3所示，氟雷拉纳、洛替拉纳、沙罗拉纳和阿福拉纳在 3种牛源性食品（牛奶、牛肉和牛肝）中的ME分别为-18.2%~12.9%、-13.0%~9.7%、-3.3%~18.0%和-8.3%~12.3%。表明基质效应在可接受范围内。

**图3:**
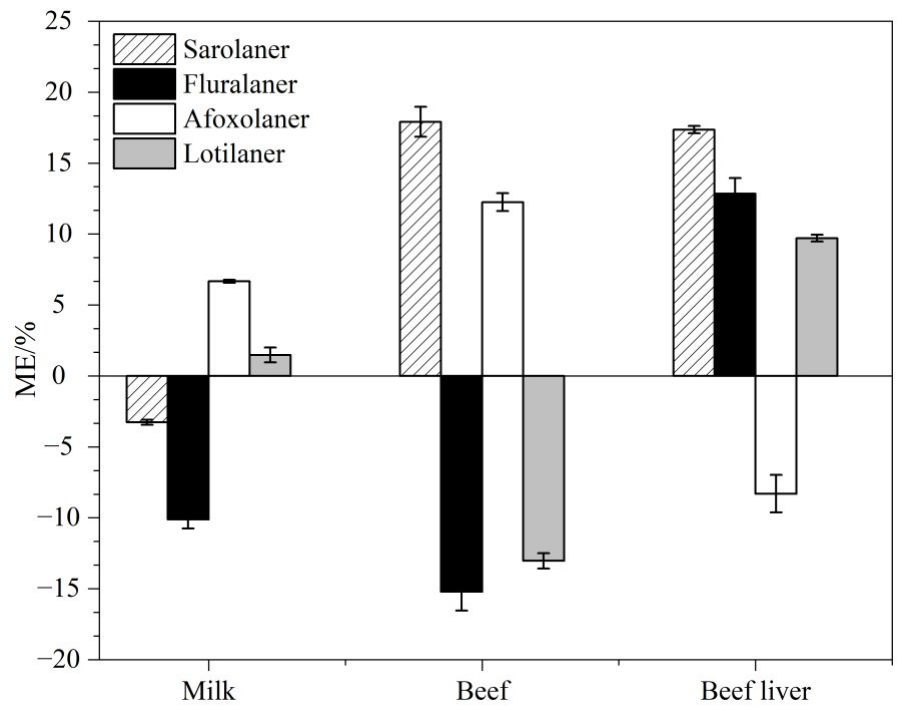
3种牛源性食品基质中4种ISOs的基质效应（*n*=3）

### 2.5 线性范围、检出限、定量限、回收率和精密度

依据GB/T 27417-2017《合格评定 化学分析方法确认和验证指南》^［21］^中的要求，对分析方法开展验证。配制系列质量浓度的4种ISOs混合标准溶液，按1.4节条件进样分析。以各目标物的峰面积为纵坐标（*y*）、质量浓度为横坐标（*x*， μg/L）绘制标准曲线。分别将信噪比（*S/N*）≥3和*S/N*≥10所对应的质量浓度作为检出限（LOD）和定量限（LOQ）。结果如表2所示，4种ISOs在各自的线性范围内具有良好的线性关系，*r*≥0.993 6。4种ISOs的LOD和LOQ分别为0.2~0.5 μg/kg和0.5~1.0 μg/kg。

**表2:** 4种ISOs的线性范围、线性方程、相关系数、检出限和定量限

Compound	Linear range/（μg/L）	Linear equation	*r*	LOD/（μg/kg）	LOQ/（μg/kg）
Fluralaner	0.5-500	*y*=4.31×10^4^ *x*+3.28×10^3^	0.9990	0.2	0.5
Lotilaner	1.0-500	*y*=4.10×10^4^ *x*+1.45×10^3^	0.9996	0.5	1.0
Afoxolaner	1.0-500	*y*=6.37×10^4^ *x*+1.71×10^4^	0.9936	0.5	1.0
Sarolaner	1.0-500	*y*=1.31×10^3^ *x*-4.81×10^3^	0.9962	0.5	1.0

*y*： peak area； *x*： mass concentration， μg/L.

在空白牛奶、牛肉和牛肝样品中分别添加低、中、高3个水平（1、2、10 μg/kg）的4种ISOs混合标准溶液，每个加标水平平行测定6次。结果如表3所示，在3个加标水平下，4种ISOs的加标回收率为67.6%~118.9%，相对标准偏差（RSD）为2.0%~20.0%。上述实验结果表明，该方法具备较高的灵敏度，同时拥有良好的准确度和精密度，适用于牛源性食品中ISOs的检测。

**表3:** 3种样品基质中4种ISOs的回收率和相对标准偏差（*n*=6）

Sample	Compound	Spiked level/（μg/kg）	Measured/（μg/kg）	Recovery/%	RSD/%
Milk	fluralaner	1	0.84	83.8	9.7
		2	1.61	80.6	8.3
		10	8.90	89.0	2.9
	lotilaner	1	0.90	90.3	11.1
		2	1.74	87.1	8.5
		10	9.24	92.4	2.2
	afoxolaner	1	0.90	89.8	7.1
		2	2.15	107.7	7.8
		10	10.75	107.5	3.2
	sarolaner	1	0.96	96.2	9.7
		2	1.73	86.4	8.8
		10	9.19	91.9	2.7
Beef	fluralaner	1	0.91	90.8	18.6
		2	1.92	96.2	11.5
		10	9.88	98.8	12.6
	lotilaner	1	0.96	95.9	20.0
		2	2.06	103.2	11.1
		10	10.24	102.4	13.2
	afoxolaner	1	0.96	95.9	19.1
		2	2.06	103.0	13.2
		10	9.63	96.3	13.3
	sarolaner	1	0.95	95.3	17.2
		2	1.88	93.9	13.7
		10	9.07	90.7	15.8
Beef liver	fluralaner	1	1.01	101.3	11.4
		2	1.56	78.1	9.4
		10	11.85	118.9	2.0
	lotilaner	1	1.00	100.2	13.5
		2	1.35	67.6	4.5
		10	11.40	114.0	4.8
	afoxolaner	1	1.17	116.8	2.2
		2	1.51	75.3	2.6
		10	9.69	96.9	6.9
	sarolaner	1	1.18	117.7	4.1
		2	1.43	71.7	4.9
		10	11.28	112.8	5.8

### 2.6 与其他方法的比较

从检测方法、样品类别、目标物数量、净化方法、提取时间以及LOD等方面，将本方法与已报道文献方法进行对比，结果如表4所示。与文献方法相比，本研究所建方法具有明显优势，如LOD更低、提取时间相对更短；同时，该方法可分析的目标物包含了4种新型ISOs。本方法能够满足对样品中多种ISOs进行定量、定性分析的需求，为后续国内牛源性食品中这4种ISOs相关标准和法规的制定提供了技术支撑与研究基础。

**表4:** 该方法与文献方法的比较

Detection method	Sample types	Compounds	Purification method	Extraction time/min	LODs/（μg/kg）	Ref.
UHPLC-MS/MS	hen tissues	fluralaner， lotilaner， afoxolaner， sarolaner	SPE （PRiME HLB column）	15	2	［^8^］
UHPLC-MS/MS	porcine liver	sarolaner	SPE （PRiME HLB column）	15	2.0	［^12^］
UHPLC-MS/MS	chicken tissues， egg	fluralaner	SPE （Oasis HLB column）	10	0.3-1.5	［^13^］
UHPLC-MS/MS	pork	fluralaner	QuEChERS （C18+PSA）	1.0	0.3	［^22^］
UHPLC-MS/MS	chicken tissues， egg	fluralaner	SPE （PRiME HLB column）	32	2.0	［^23^］
UHPLC-Q/Trap MS	milk， beef， beef liver	fluralaner， lotilaner， afoxolaner，sarolaner	SPE （PRiME HLB column）	10	0.2-0.5	this work

### 2.7 实际样品的检测

为评估该方法的有效性，采用本文所建分析方法对郑州市3个不同地区的市售牛肉、牛奶和牛肝共计9份样品展开分析检测。检测结果经MRM-IDA-EPI谱库检索进一步确证，最终结果表明，所有样品中均未检出4种新型ISOs。

## 3 结论

本研究将固相萃取技术与UHPLC-Q/Trap MS技术结合，利用MRM-IDA-EPI扫描模式，建立了一种可同时测定牛源性食品（牛奶、牛肉和牛肝）中4种新型ISOs（氟雷拉纳、洛替拉纳、沙罗拉纳和阿福拉纳）的分析方法。研究对样品前处理方法和仪器分析条件进行了优化，有效降低了样品中复杂基质对分析结果产生的干扰。同时，结合MRM-IDA-EPI谱库检索技术，提高了ISOs定性分析的准确性，有效避免了假阳性结果的出现。本方法操作简便、检出限和定量限较低、线性范围较宽、准确度和精密度良好，能够满足GB/T 27404-2008所规定的质量控制要求。该方法可用于复杂动物源性食品中新型ISOs残留的分析，为动物源性食品中新型ISOs残留的监管提供了可靠的技术支撑。
